# Sinularin exerts anti-tumor effects against human renal cancer cells relies on the generation of ROS

**DOI:** 10.7150/jca.31232

**Published:** 2019-08-28

**Authors:** Qi Ma, Xiang-Yu Meng, Ke-Rong Wu, Jian-Zhou Cao, Rui Yu, Ze-Jun Yan

**Affiliations:** 1 Translational Research Laboratory for Urology, the Key Laboratory of Ningbo city, Ningbo First Hospital, The Affiliated Hospital of Ningbo University. #59 Liuting street, Ningbo, ZJ, 315010, China.; 2 Department of Urology, Ningbo First Hospital, The Affiliated Hospital of Ningbo University. #59 Liuting street, Ningbo, ZJ, 315010, China.; 3 School of Medicine, Ningbo University. #818 Fenghua Road, Ningbo, ZJ, 315211, China.; 4 Department of Biochemistry and Molecular Biology, Zhejiang Key laboratory of Pathophysiology, School of Medicine, Ningbo University. #818 Fenghua Road, Ningbo, ZJ, 315211, China.

**Keywords:** Sinularin, apoptosis, ROS, MAPKs, PI3K/Akt/mTOR pathway

## Abstract

Sinularin, a soft corals-derived natural product, exerts anti-tumorigenic activity in various types of human cancer cells. However, the action of Sinularin and its mechanism in renal carcinoma is not well understood. In the current study, we demonstrated that Sinularin inhibited the viability of human renal cancer cells 786-O and ACHN in a dose- and time-dependent manner, but did not show significant toxicity against non-malignant HRCEpic cells. Cell cycle analysis revealed that Sinularin induced G2/M arrest significantly. In addition, Sinularin could induce apoptosis in cells along with caspase-3/-9 activation, release of mitochondrial proteins, up-regulation of pro-apoptotic Bcl-2 family proteins and inhibition of anti-apoptotic Bcl-2 family proteins. Sinularin could also repress the activation of PI3K/Akt/mTOR signaling pathway. Moreover, Sinularin triggered the activation of MAPKs and p38 activation was essential for the anti-tumor effect of Sinularin. The generation of ROS (reactive oxygen species) was critical for Sinularin-induced apoptosis since ROS scavenger NAC (N-acetyl cysteine) could block the Sinularin-triggered apoptosis. In conclusion, all the results indicated that Sinularin may be applied as a therapeutic natural agent for human renal cancer.

## Introduction

Renal cell carcinoma (RCC), one of the most common diagnosed cancers in urological system, accounts for about 3% of all cancers in adults worldwide 1. Among all the subtypes of RCC, clear cell renal cell carcinoma (ccRCC) is the most common subtype and is responsible for around 85% of all RCC cases 2. Approximately 30% of RCC patients have metastatic lesions that are detected at diagnosis 3. Metastatic RCC patients' prognosis is poor, and the median survival can be less than 12 months 4. Traditional therapeutics such as chemotherapy, radiotherapy and immunotherapy have limited effects on RCC. Although novel small molecule-targeted anti-tumor agents such as sorafenib and sunitinib are promising, several side effects have been raised during recent trials, i.e. the risk of adverse events and unwanted efficacy-loss 5, 6. Therefore, searching for novel therapeutic strategies against RCC cancer becomes increasingly vital.

Currently, plenty of anti-cancer agents trigger cell death via inducing apoptosis. There are two apoptotic pathways: the extrinsic and intrinsic pathway. The former pathway is mainly mediated by death receptors, characterized as caspase-8 activation. The latter pathway is mainly mediated by mitochondria, with the features of mitochondrial membrane permeabilization and reactive oxygen species (ROS) release 7. There are still other anti-cancer agents which target cells proliferation pathways, involving mitogen activated protein kinase (MAPK) and phosphatidylinositol 3-kinase (PI3K) / serine-threonine kinase (Akt) pathway 8, 9. Based on these concepts, researchers are keep looking for new therapeutic agents and approaches for RCC.

**Figure 1:**
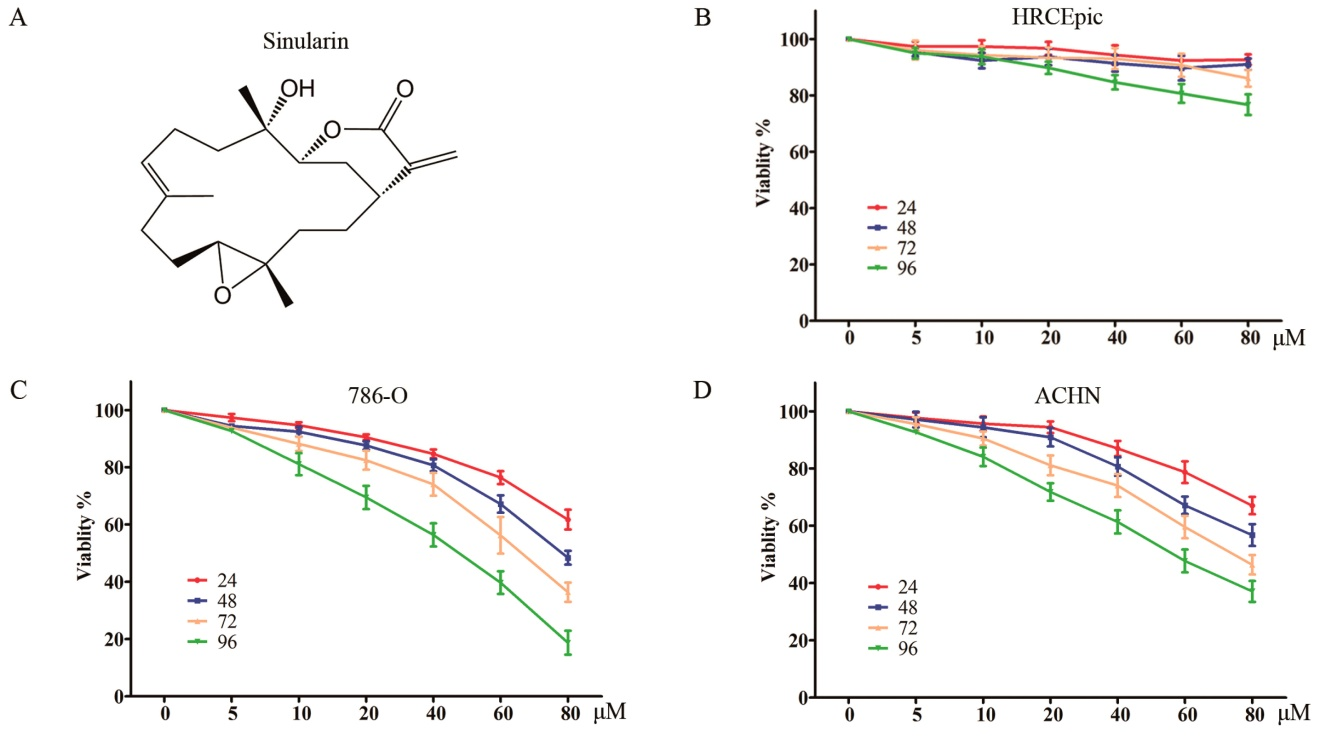
** Sinularin inhibits the viability of human RCC cells.** (A) Chemical Structure of Sinularin. (B), (C) and (D) HRCEpiC, 786-O and ACHN cells were treated with indicated concentrations of Sinularin (5, 10, 20, 40, 60 and 80 μM) for 24, 48, 72 and 96 h, then cell viabilities were measured. The data are expressed as the mean±SD from three independent experiments.

Recently, natural agents are becoming increasingly appreciated as an effective strategy of cancer chemoprevention due to its relatively low toxicity. Sinularin (Figure 1A) is a natural product that can be purified from cultured soft coral, *Sinularia flexibilis*. It has been found that Sinularin possesses anti-tumor effect against various human cancer cells such as oral cancer, hepatocellular cancer, gastric cancer, and melanoma cancer 10-13. However, the effect of Sinularin on RCC remains to be clarified. In the present study, we demonstrated that Sinularin was an anti-tumor natural compound against human RCC cells. It could induce apoptosis which relied on the generation of intracellular ROS. Moreover, Sinularin inhibited the PI3K/Akt/mTOR and activated the MAPKs signaling pathways. The results of our study additionally suggested the potential benefits of the use of Sinularin in clinical application.

## Materials and methods

### Cell culture and chemicals

Human renal cancer cell lines (786-O, ACHN) were purchased from American Type Culture Collection (ATCC) (VA, USA). Non-malignant HRCEpiC was obtained from Cell Bank, Type Culture Collection, Chinese Academy of Sciences (Shanghai, China). These cells were culture in RPMI 1640 medium (Hyclone, USA) supplemented with 10% (v/v) fetal bovine serum (Hyclone), 100 IU/ml penicillin (Hyclone) and 100 μg/ml streptomycin (Hyclone) at 37°C in a humidified atmosphere with 5% CO_2_. When reaching 70% confluence, cells were treated with control or various doses of Sinularin (Sinularin was purchased from Jianshi biotech, Zhejiang, China) for various times. At the indicated time points, cells were trypsinized and conducted to the experiments of cell proliferation, cell cycle and cell apoptosis determination. Pan-caspase inhibitor (Z-VAD-FMK) and Caspase-3 inhibitor (Z-DEVD-FMK) were purchased from Selleck (CA, USA). 2',7'-dichlorofluorescein-diacetate (DCFH-DA), N-acetyl cysteine (NAC) and DMSO were purchased from Sigma (St Louis, MO, USA). 3-(4,5-dimethylthiazol-2-yl)-5-(3-carboxymethoxyphenyl)-2-(4-sulfophenyl)-2H-tetrazolium (MTS) was purchased from Promega (Madison, Wisconsin, USA). All other routine chemicals were obtained from Sigma (St Louis, MO, USA).

### Cell viability assay

Cell viability was determined by MTS assay. Cells were seeded at a density of 1.0 × 10^4^/mL with 0.1 mL in 96-well plates, and treated with or without Sinularin for different time. After treatment, 20 μl MTS were added to each well, and the cells were incubated for another 4 h. Absorbance was measured by a microplate reader at 490 nm. The cell viability were calculated using the formula: cell viability (%) = 100×(OD_sample_ - OD_blank_)/(OD_control_ - OD_blank_).

### Cell cycle analysis

Cell cycle was determined by DNA content which was stained by propidium iodide (PI), and detected using flow cytometry. After treatment, cells were harvested by trypsinization and stained with PI (MultiSciences, Hangzhou, China) for 30 min in dark at room temperature. The cell cycle distribution was evaluated using flowcytometry (BD Bioscience, USA).

### Apoptosis analysis

After treatment, cells were harvested and stained with an Annexin V-FITC/PI Apoptosis Detection Kit (MultiSciences) according to the manufacturer's guide. The apoptosis was evaluated using flowcytometry (BD Bioscience), and data were analyzed using FlowJo V10.

### Western blot analysis

After treatment, cells were rinsed in PBS and extracts were prepared in RIPA lysis buffer (50 mM Tris HCl pH 7.5, 1 mM EDTA, 1 mM EGTA, 1 mM Na_3_VO_4_, 10 mM Na β-glycerophosphate, 50 mM NaF, 5 mM Na Pyrophosphate, 270 mM sucrose and 1% Triton X-100) supplemented with protease inhibitor cocktail (Roche, Mannheim, Germany). 30 μg of extracts were fractioned by SDS-PAGE and transferred to PVDF membrane (Invitrogen, USA). Proteins were probed with primary antibodies (Cell signaling technology, USA) and detected using corresponding secondary antibodies (Cell signaling technology). Blots were visualized by chemiluminescence (Millipore, Billerica, USA).

### Subcellular fraction

The cellular fractions were isolated using a special cytosolic and mitochondrial fraction kits (Beyotime, Shanghai, China), according to the manufacturer's guide. Briefly, after treatment cells were harvested and isolation reagents were added incubated on ice for 30 min followed by centrifugation at 1,500 rpm for 5 min. Supernatants were further centrifuged at 12,000 rpm for 10 min. The mitochondrial fraction was considered in the pellets, while the cytosolic fraction was considered in the supernatant.

### Measurement of ROS

The levels of intracellular ROS in cells were examined using the fluorescent probe DCFH-DA. Briefly, after treatment cells were washed twice in PBS and loaded with 10 μM DCFH-DA probes for 30 min in dark. The levels of ROS were indicated by DCF fluorescence, which were measured using a flow cytometric analyses (Becton Dickinson, San Jose, CA). Data were analyzed using FlowJo V10.

### Statistical analysis

Variance between the control and tested groups were analyzed using one-way analysis of variance (ANOVA) procedure followed by Tukey's post hoc test. Variance between two groups was assessed using student* t* test. Statistical significances were set as *P* < 0.05, and all the data were expressed as mean ± standard deviation (SD).

## Results

### Sinularin reduces cell viability of human renal cancer cells

The anti-viability effects on RCC cells of Sinularin were evaluated by MTS assay. Two renal cancer cell lines (786-O and ACHN) and non-malignant human renal epithelial cells (HRCEpiC) were treated with various doses of Sinularin (5, 10, 20, 40, 60 and 80 μM) for different time (24, 48, 72 and 96 h). As indicated in Figure 1 C and D, Sinularin significantly decreased the viability of human renal cancer cells ACHN and 786-O in a dose- and time-dependent manner. However, Sinularin had little effect on the viability of HRCEpiC cells (Figure 1B). These results indicated that Sinularin shows selectively anti-tumor activity in human renal cancer cells. The IC50 of Sinularin in renal cancer cells were listed in Table 1. Since 786-O cell was more sensitive to Sinularin than ACHN cells, it was chosen in the following investigations.

### Sinularin induces cell cycle arrest at the G2/M phase

**Figure 2:**
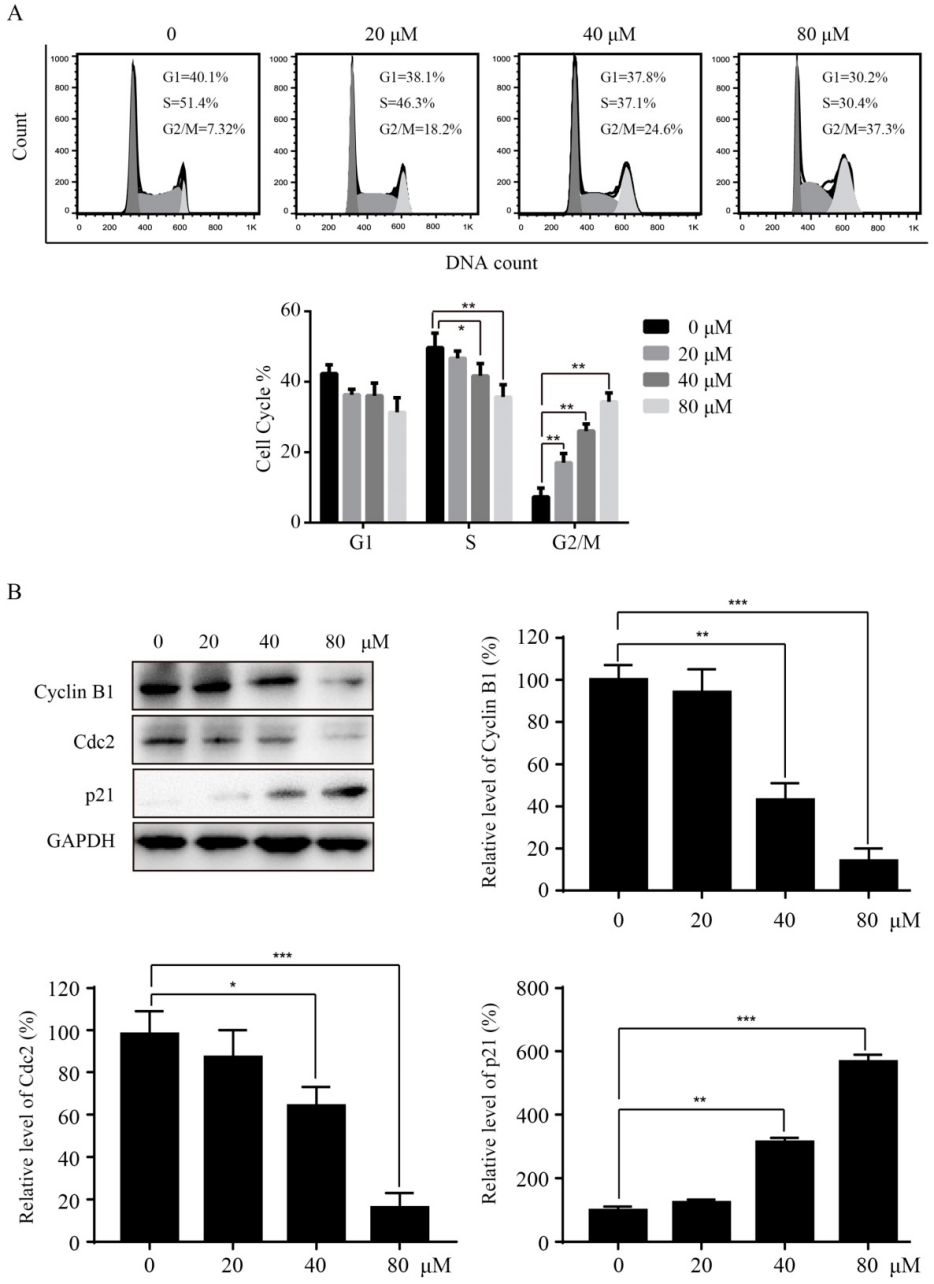
** Sinularin induces cell cycle arrest at G2/M phase in 786-O cells.** (A) 786-O cells were treated with different doses of Sinularin (20, 40, 80μM) for 24 h, and cell cycle were analyzed by flow cytometry. (B) 786-O cells were treated with different doses of Sinularin (20, 40, 80μM) for 24 h, and cellular lysates were subjected to western blot analysis with the indicated antibodies. The data are expressed as the mean±SD from three independent experiments.*P<0.05, **P<0.01, ***P<0.001.

To investigate the mechanisms of Sinularin in reducing cell viability, we tested whether Sinularin affected the cell cycle progression in 786-O cells. After treatment with the indicated concentration of Sinularin (20, 40, 80 μM) for 24 h, 786-O cells were stained with PI and analyzed for DNA content by flow cytometry. As indicated in Figure 2A, treatment with Sinularin led to cell cycle arrest at the G2/M phase in a dose-dependent manner, while there was a decrease in S phase. To further unveil the mechanism of cell cycle arrest, we detected the levels of cell cycle-regulated proteins by western blot. As shown in Figure 2B, Sinularin treatment decreased the expression of Cyclin B1, Cdc2 and increased the expression of p21. These results indicate that cell viability repressed by Sinularin may be due to cell cycle arrest.

### Sinularin induces apoptosis in renal cancer cells

**Figure 3:**
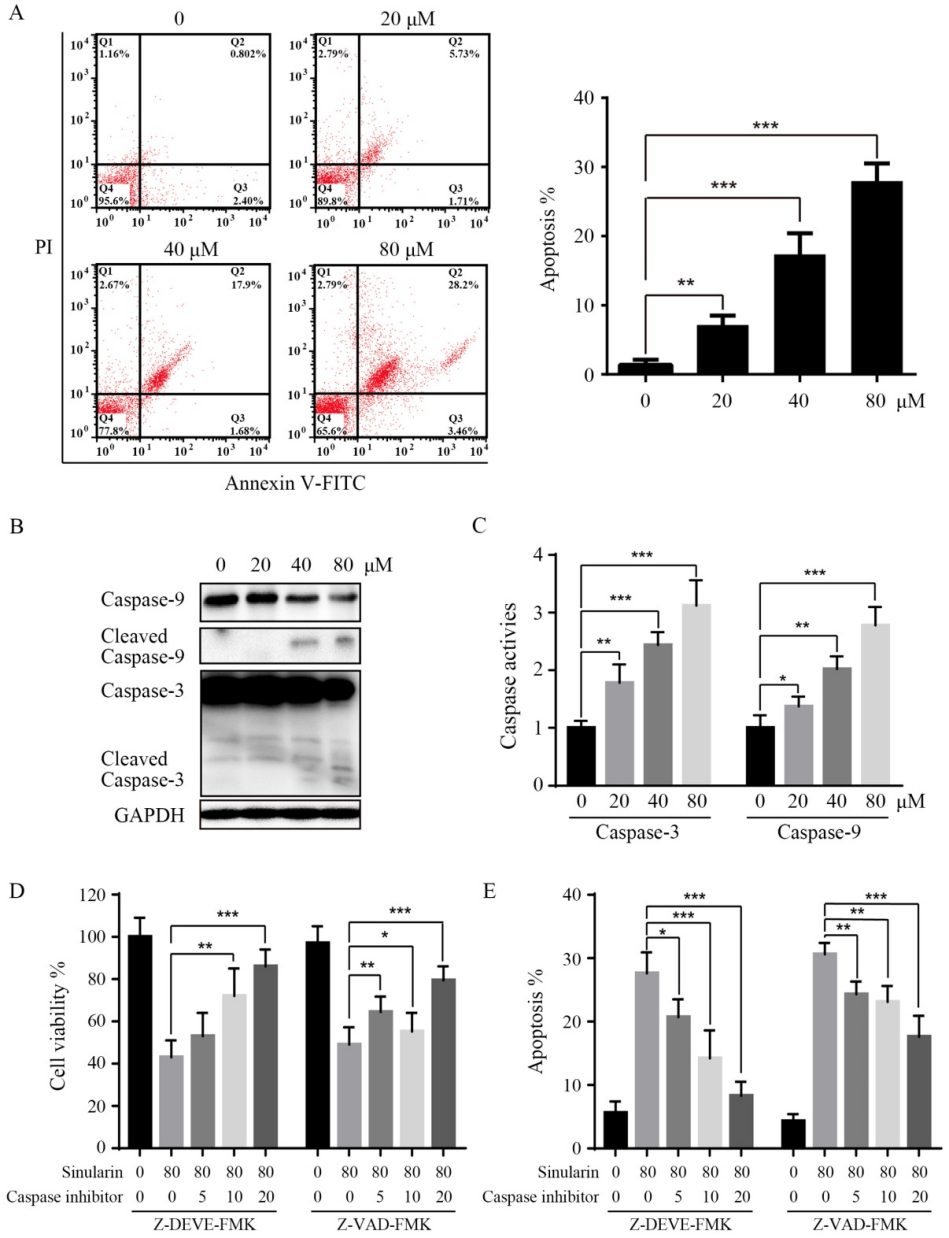
** Sinularin induces apoptosis in 786-O cells.** After treated with various doses of Sinularin (20, 40, 80μM) for 24 h. (A) Cell apoptosis was measured by flow cytometry. (B) Cellular lysates were subjected to western blot analysis with indicated antibodies. (C) Caspases activities were assayed. (D) Cells were treated with Sinularin alone (80 μM) or in combination with Z-DEVD-FMK or Z-VAD-FMK for 24 h and viability were measured. (E) Cells were treated with Sinularin alone (80 μM) or in combination with Z-DEVD-FMK or Z-VAD-FMK for 24 h and apoptosis were measured. The data are expressed as the mean±SD from three independent experiments.*P<0.05, **P<0.01, ***P<0.001.

In order to further elucidate the mechanisms of Sinularin in repressing cell viability, we examined the apoptosis by flow cytometry, and the Annexin V/PI patterns of Sinularin-treated 786-O cells were analyzed (Figure 3A). As indicated, after the treatment of Sinularin for 24 h, the percentages of Annexin V/PI positive cells were increased in a dose-dependent manner in 786-O cells. Then the status of caspases after the treatment of Sinularin was examined, cleaved caspase-3 and cleaved caspase-9 were increased in a dose-dependent manner (Figure 3B). Similarly, activities of caspase-3 and caspase-9 were also increased after the treatment (Figure 3C). To further investigate the role of caspases in Sinularin-induced apoptosis, cells were pretreated with pan-caspase inhibtor (Z-VAD-FMK) and specific caspase inhibitor for caspase-3 (Z-DEVD-FMK) for 1 h and then incubated with Sinularin for 24h. As shown in Figure 3D and 3E, both pan-caspase inhibitor Z-VAD-FMK and caspase-3 inhibitor Z-DEVD-FMK can blunt the cytotoxic effects of Sinularin. Taken together, these findings indicate that Sinularin may induce apoptosis relies on the activation of caspases.

### Sinularin activates the intrinsic apoptotic signaling pathway via modulation of Bcl-2 proteins

**Figure 4:**
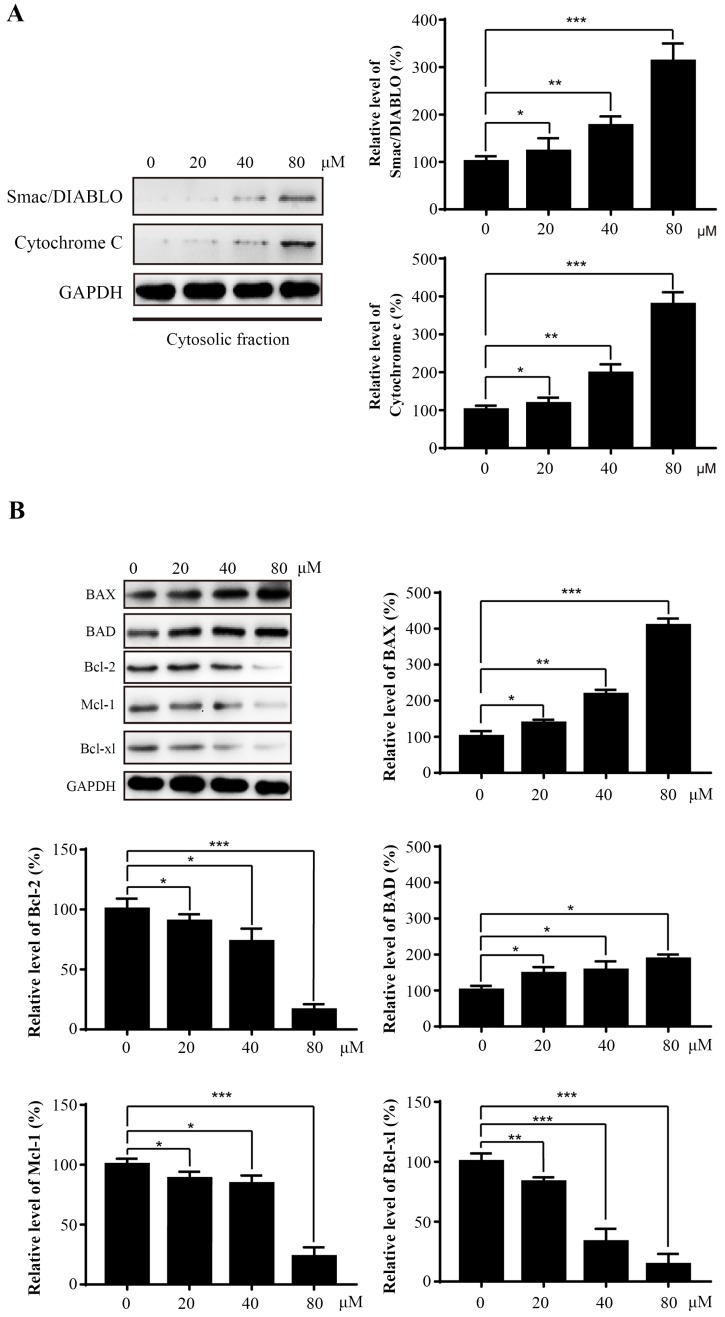
** Sinularin induces the release of mitochondrial proteins into cytosol and affects the expression of Bcl-2 proteins.** 786-O cells were treated with various concentrations of Sinularin (20, 40, 80μM) for 24 h. (A) Cellular cytosolic fraction was subjected to western blot analysis with the indicated antibodies. (B) Total cellular lysates were subjected to western blot analysis with the indicated antibodies. The data are expressed as the mean±SD from three independent experiments.*P<0.05, **P<0.01, ***P<0.001.

Mitochondria play an essential role in the process of apoptosis. To further explore the potential mechanism of Sinularin-induced apoptosis, several mitochondria-related proteins were examined by western blot. An increase of mitochondrial proteins Smac/DIABLO and Cytochrome c in the cytosol were detected after exposure to Sinularin (Figure 4A). The Bcl-2 family proteins are critical regulators of mitochondrial-mediated apoptosis. Therefore, we asked whether the levels of Bcl-2 family proteins were affected after the treatment of Sinularin as well. The results demonstrated that the anti-apoptotic proteins like Bcl-2, Mcl-1 and Bcl-xl were decreased and the pro-apoptotic proteins like Bax and Bad were increased in a dose-dependent manner after the treatment of Sinularin.

### Sinularin represses PI3K/AKT/mTOR and activated MAPK pathways

**Figure 5:**
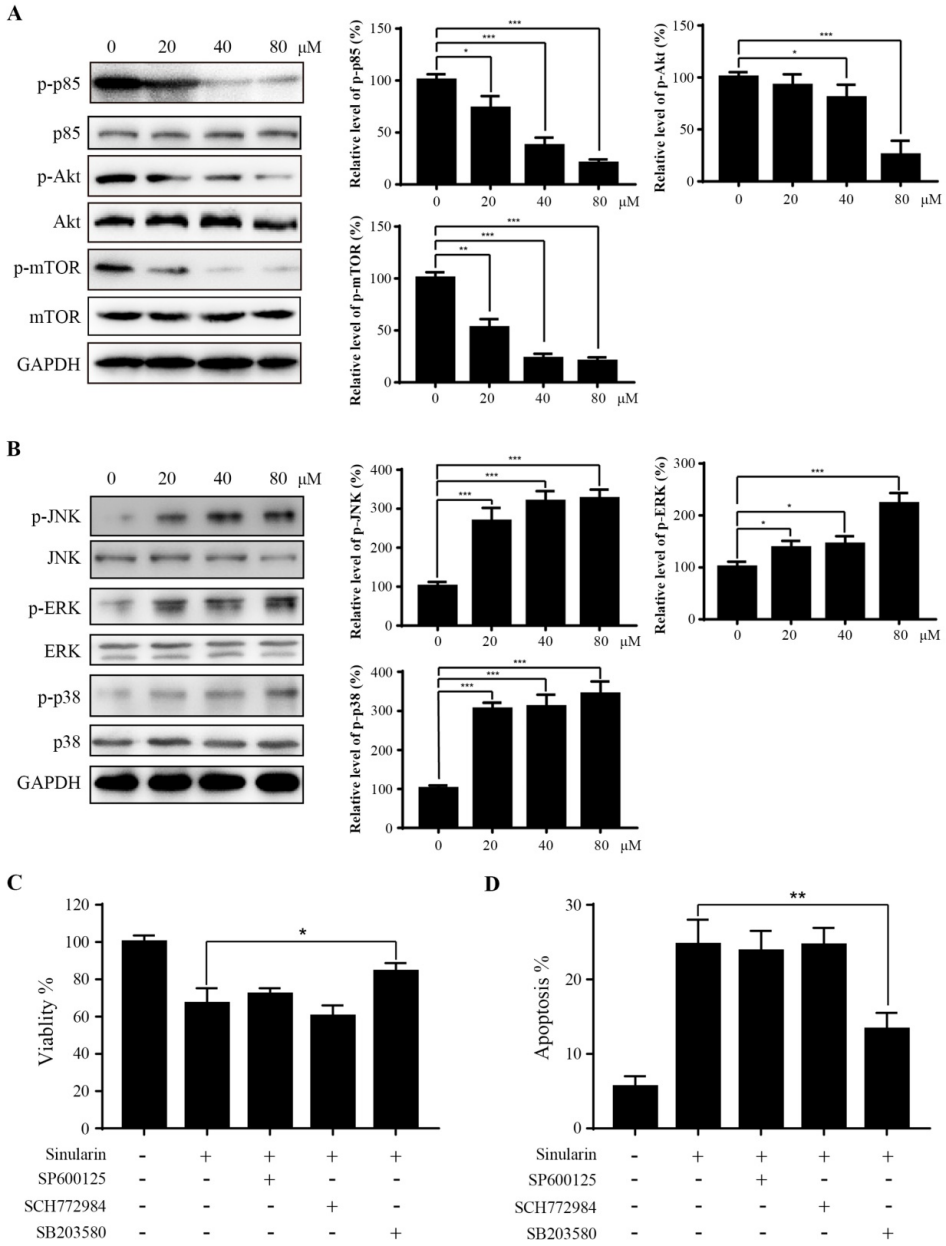
** Sinularin inhibits PI3K/Akt and activates MAPKs pathways.** (A) and (B) 786-O cells were treated with various concentrations of Sinularin (20, 40, 80 μM) for 24 h. Then total cellular lysates were subjected to western blot analysis with the indicated antibodies. (C) 786-O cells were treated with Sinularin (80 μM) in the presence of SP600125 or SCH772984 or SB203580, then the cell viability was measured. (D) 786-O cells were treated with Sinularin (80 μM) in the presence of SP600125 or SCH772984 or SB203580, then the cellular apoptosis was measured. The data are expressed as the mean±SD from three independent experiments.*P<0.05, **P<0.01, ***P<0.001.

PI3K/Akt/mTOR is a signaling pathway involved in various biological activities such as cell cycle, growth, metabolism and tumorigenesis 14. To evaluate the effect of Sinularin on PI3K/Akt/mTOR pathway, the p-PI3K p85, PI3K p85, p-Akt, Akt, p-mTOR and mTOR were detected by western blot assays. When treated with Sinularin, the amount of p-PI3K p85, p-Akt and p-mTOR were significantly decreased compared with the control. However, the level of total PI3K p85, Akt and mTOR were not affected (Figure 5A). These findings suggest that PI3K/Akt/mTOR pathway is involved in Sinularin-induced apoptosis.

MAPK signaling pathways (p38 and JNK) are found associated with apoptosis. We asked whether MAPK signal pathways are also involved in Sinularin induced apoptosis. The expression of phosphorylated MAPKs (p38 and JNK) were increased after the treatment of Sinularin, while the total JNK and p38 levels were not affected (Figure 5B). Interestingly, ERK signaling was also activated after Sinularin treatment (Figure 5B). To further characterize the function of MAPKs in Sinularin-induced apoptosis, three different specific inhibitors were tested. As indicated in Figure 5C and D, the increasing cell viability and decreasing apoptosis were observed after treatment with SB203580 (p38 inhibitor) in the Sinularin-treated cells. By contrast, neither SCH772984 (ERK inhibitor) nor SP600125 (JNK inhibitor) has the similar effects. Since SB203580 is a specific p38 inhibitor, therefore Sinularin may exert its anti-tumor effects via the activation of p38 pathway.

### The effects of ROS in apoptosis induced by Sinularin

**Figure 6:**
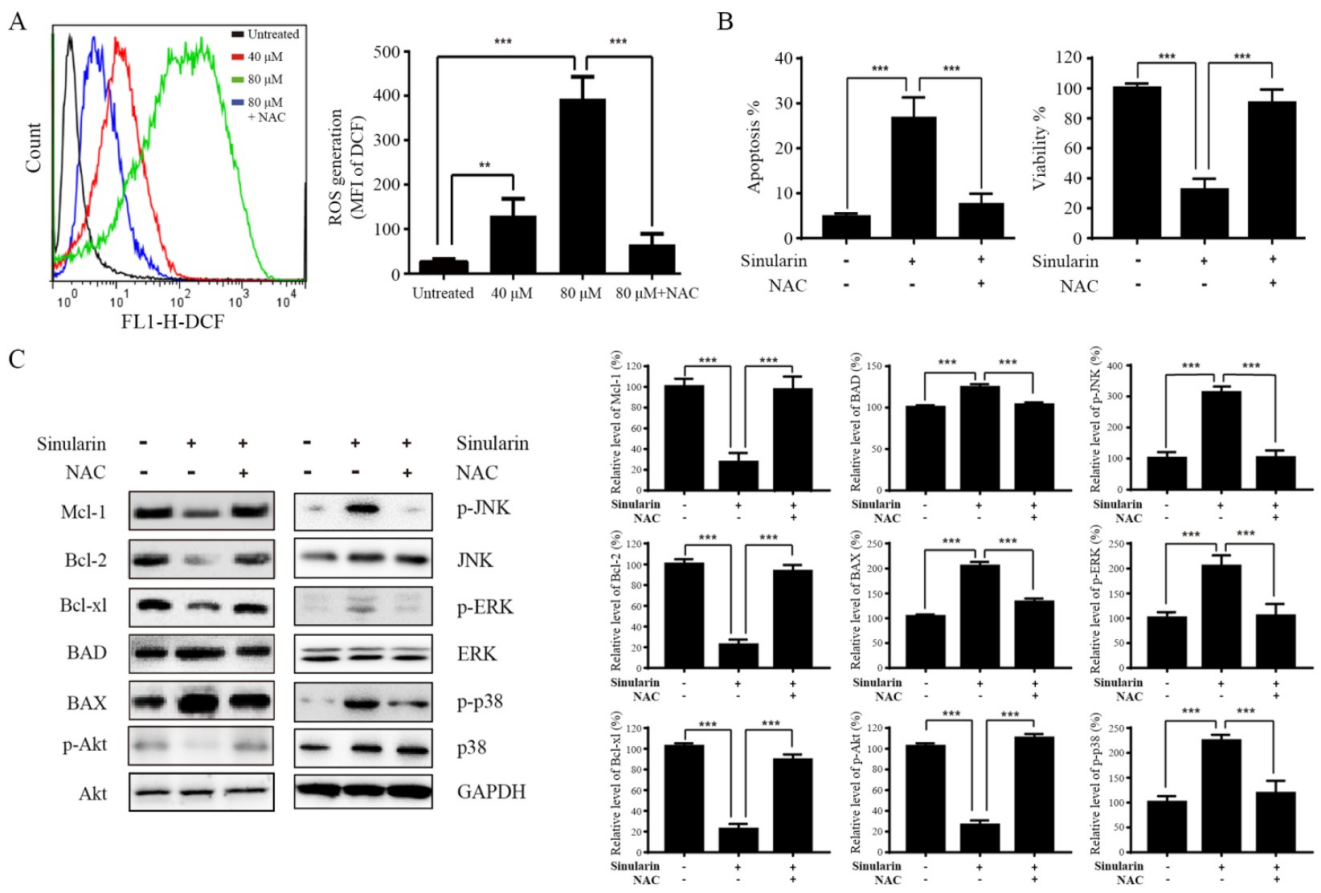
** Sinularin exterts anti-tumor effects relies on the generation of ROS.** (A) 786-O cells were treated with different doses of Sinularin (40, 80 μM) for 24 h. Intracellular ROS levels were measured by flow cytometry. (B) 786-O cells were treated with Sinularin (80 μM) with or without NAC (100 μM) for 24 h, then cellular apoptosis and viability were measured. (C) 786-O cells were treated with Sinularin (80 μM) with or without NAC (100 μM) for 24 h, then total cellular lysates were subjected to western blot analysis with indicated antibodies. The data are expressed as the mean±SD from three independent experiments.*P<0.05, **P<0.01, ***P<0.001.

ROS generation is an essential regulator in a variety of cellular process, such as apoptosis, inflammation, autophagy and metabolism via alteration of various signaling pathways 15. Enhancement of DCF fluorescence in 786-O cells after Sinularin treatment was detected compared to the untreated control group (Figure 6A). Moreover, Sinularin-induced ROS could be reduced by N-acetyl cysteine (NAC) which is a ROS scavenger (Figure 6A). In addition, Sinularin-triggered apoptosis and Sinularin-reduced cell viability could be reversed by NAC (Figure 6B). Next, we assessed the relationship between ROS accumulation, Bcl-2 family members, PI3K/Akt and MAPKs signaling pathways. As shown in Figure 6C, NAC treatment could abrogate the Sinularin-induced repression of anti-apoptotic Bcl-2 family members such Mcl-1, Bcl-2, and Bcl-xl. In addition, the up-regulation of BAD and BAX were also inhibited after NAC treatment. Meanwhile, application of NAC abolished Sinularin-induced reduction of p-Akt. Moreover, NAC application reduced Sinularin-induced activation of p38 (Figure 6C). Thus, we believe that Sinularin exerts its anti-tumor effect relies on the inhibition of PI3K/Akt and activation of p38 which are all relied on the generation of ROS.

## Discussion

Despite significant progress in the detection and management of RCC, there are little effective adjuvant therapies for RCC, although different combinations have been tested clinically 5. Thus, it is needed to implement biologically safe and effective natural products as anti-tumor and chemo-preventive drugs. Sinularin is an active compound isolated from the cultured soft coral *Sinularia flexibilis* that has been suggested to have anticancer effects. The present study was designed to investigate the anti-tumor effects against RCC cells and its underline mechanisms. The results showed that Sinularin reduced cell viability, induced G2/M arrest and apoptosis in 786-O cells. The anti-tumor effects of Sinularin were associated with p38 and PI3K/Akt-mediated pathways which were affected by the production of ROS.

In the present study, we found that Sinularin significantly inhibited the proliferation of renal cancer cells 786-O and ACHN, but it exerted little toxicity toward non-malignant HRCEpiC cell. Therefore, Sinularin might represent a promising therapeutic agent against RCC. Sinularin induced accumulation of G2/M phase. Similarly, previous studies have also indicated that the anti-tumor effects of Sinularin were related to G2/M cell cycle arrest in various cancer cells like hepatocellular carcinoma cells and oral cancer cells 12, 13. The G2/M phase progression is regulated by cyclin B1 and CDK1 complex, which are controlled by cdc25c. Here we found that Sinularin significantly decreased the levels of cyclin B1 and CDK1 while increased the level of p21. These findings suggest that Sinularin induced G2/M arrest might be associated with down-regulation of cyclin B1 and CDK1 complex and up-regulation of p21.

Apoptosis plays an essential role in regulating growth, immune response, and clearing redundant or abnormal cells to maintain tissue homeostasis 16. The apoptosis of 786-O cells induced by Sinularin was also observed by using PI and Annexin V double staining. Apoptosis can be executed through either extrinsic pathway and/or intrinsic pathway which is also known as mitochondrial pathway. In this study, Sinularin activated caspase-3 and caspase-9 were observed. Furthermore, pan-canspase and caspase-3 inhibitors could inhibit the Sinularin-induced cell apoptosis. Previous study found that Sinularin-induced apoptosis is associated with intrinsic/mitochondrial pathway which is regulated by the Bcl-2 family proteins 13. Our results showed that Sinularin enhanced the expression of pro-apoptotic Bcl-2 proteins (BAD and BAX) while repressed the expression of anti-apoptotic Bcl-2 proteins. Furthermore, the release of cytochrome c and Smac/DIABLO into cytosol were observed as well. These data suggest that Sinularin induced apoptosis via the intrinsic pathway in a caspase-dependent manner.

It is well documented that MAPK signaling pathways (ERK, p38, JNK) are involved in mediating various cellular process including apoptosis. Therefore, we examined the phosphorylation status of MAPKs to elucidate the molecular mechanisms that participated in Sinularin-induced apoptosis. Our data showed that JNK, p38 and ERK are all phosphorylated after the treatment of Sinularin. However, the cell death was blocked by p38 inhibitor only, suggesting that p38 was involved in Sinularin-induced apoptosis. Moreover, our study demonstrated that Sinularin dephosphorylated Akt. PI3K/Akt signaling pathway is frequently dysregulated in various cancers, and Akt is a downstream effector of PI3K that regulates many biological processes 14. We found that Sinularin treatment strikingly reduced phosphorylation of PI3K and Akt, meanwhile the mTOR was significantly repressed. Therefore, our results suggest, at least partially, that Sinularin induces apoptosis via down-regulation of PI3K/Akt/mTOR signaling pathway. This finding is in line with a previous report in which Sinularin could inhibit PI3K/Akt in gastric cancer cells 11. Interestingly, 13-acetoxysarcocrassolide, another compound isolated from soft coral *Sarcophyton crassocaule*, also induced apoptosis via repression of PI3K/Akt pathway 17.

ROS activates different cellular signal pathways, which regulate different biological processes such as cell cycle progression, migration, invasion and cell death 15. Many reports have shown that intracellular ROS production has vital roles in MAPK activation and PI3K/Akt repression during apoptosis 18. It has been suggested that Sinularin inhibited tumor cells relies on the generation of ROS 12. Similarly, we found that Sinularin notably induced ROS production. In addition, scavenge of ROS generation by NAC could abrogate the activation of MAPKs, inhibition of PI3K/Akt, as well as apoptosis induced by Sinularin.

In summary, our study indicated the anti-tumor effect of Sinularin against RCC cells. Sinularin markedly suppresses the growth and induces apoptosis as well as activation of MAPKs and repression of PI3K/AKT pathways, which are dependent on ROS generation. Considering Sinularin also possesses anti-inflammation activity 19. Thus, Sinularin might be a novel therapeutic strategy for management of human RCC.

## Figures and Tables

**Table 1:** The IC50 values of Sinularin in renal cancer cells

Cells	24 h	48 h
786-O	124.4 μM	86.63 μM
ACHN	132.5 μM	98.56 μM
